# Pediatric forearm fractures with in situ intramedullary implants

**DOI:** 10.1007/s11832-016-0746-4

**Published:** 2016-06-08

**Authors:** Brian A. Kelly, Benjamin J. Shore, Donald S. Bae, Daniel J. Hedequist, Michael P. Glotzbecker

**Affiliations:** Department of Orthopaedic Surgery, Boston Children’s Hospital and Harvard Medical School, 300 Longwood Avenue, Boston, MA 02115 USA

**Keywords:** Pediatric, Forearm, Fracture, In situ, Implants

## Abstract

**Purpose:**

The purpose of this investigation is to present our institutional experience with fractures of the pediatric forearm with in situ intramedullary nails.

**Methods:**

Six patients treated at our institution for forearm fracture with in situ intramedullary implants between 2004 and 2013 were reviewed. Patient demographics, injury and radiographic characteristics, method of treatment, time to union, and complications were collected from the medical record.

**Results:**

485 patients with forearm fractures were treated with intramedullary implants and six patients presented with a fracture with in situ implants (1.2 %). Fractures in all six patients resulted from a second traumatic event after radiographic healing but before implant removal at a mean of 13.0 months from the initial procedure. One patient had an adequately aligned fracture and was treated with casting without reduction. The remaining five patients (83 %) returned to the operating room for treatment. Two patients underwent rod removal and placement of new intramedullary implants, and two patients were treated with rod removal and plating without attempt at closed reduction. One patient underwent closed reduction in the operating room with successful re-bending of the radial implant and replacement of the ulna implant. All patients went on to uncomplicated radiographic union at a mean 3.6 months.

**Conclusions:**

The incidence of fracture of pediatric forearm with in situ intramedullary implants is low. This rare complication can be treated by several different methods, including revision TENS placement, revision to plate fixation, or in situ bending of rods, with the expectation for successful uncomplicated union.

## Introduction

Fractures of the forearm are one of the most common injuries seen in childhood [1, 2]. Most of these fractures can be treated by closed means with reduction and cast immobilization; however, unstable or open injuries often require surgical treatment to maintain adequate alignment [3, 4]. Intramedullary (IM) fixation with titanium elastic nails (TENS) or Kirchner wires (K-wires) has emerged as the most common method for fixation of forearm fractures in skeletally immature patients [5, 6]. While practices regarding removal of these implants vary considerably, implant removal is typically performed 6 months to a year after the index procedure [7–9]. Refractures occur in 4–8 % of patients treated non-operatively, which has historically dictated the timing of removal of these implants [10, 11].

There are several case reports in the literature of fractures that occur after radiographic healing but prior to removal of the IM implants as well as description of this complication in larger series; however, no conclusions can be drawn about the incidence of this complication, optimal treatment after refracture with in situ implants, or possible risk factors leading to refracture [12–21]. The purpose of this investigation therefore was to review our institutional experience with forearm fractures with in situ IM fixation and present the characteristics, treatment, and outcomes of these complications, as well as to estimate the frequency of this complication. We hypothesize that these patients will require a second operative procedure but will go on to uncomplicated union.

## Methods

### Retrospective review

After institutional review board approval, a retrospective investigation of all children 1–18 years of age treated with IM fixation for fractures of the forearm bones between 2004 and 2013 at a single tertiary care pediatric hospital was performed. Initial search demonstrated 485 patients with fractures of the forearm bones treated with intramedullary implants. Patients who suffered any fracture of the forearm bones in the same arm prior to removal of the intramedullary implants were included in the review. Procedures were performed by board certified, fellowship trained pediatric orthopaedic surgeons. All fractures of the radius and/or ulna treated with IM implants (Including Monteggia fractures, radial neck fractures, etc.) were included. Patients were excluded if adequate records, imaging, or clinical follow-up were unavailable. Data on demographics, mechanism and type of injury, radiographic characteristics, type of treatment and surgical technique, time to union, and complications was collected. Specific complications of interest included: infection, refracture, non or mal-union, tendon rupture, nerve injury, implant migration through skin, loss of reduction, hypertrophic granuloma, loss of functional motion, and other complications.

### Surgical technique

Technique for index procedure varied by surgeon and type of fracture. In all cases, an attempt was made at closed reduction and percutaneous insertion of titanium flexible nails (Titanium Elastic Nails, Synthes, West Chester, PA) or stainless steel Kirschner-wires (K-wires) based on surgeon preference. Irrigation and debridement was performed for all open fractures, and limited open reduction was performed as necessary to obtain alignment and pass the nails. All fixation of the ulna was performed in an antegrade fashion with a starting point at the olecranon tip or proximal lateral metaphysis based on surgeon preference. The olecranon tip starting point was often selected due to the ease of insertion and removal of the implant. Single bone fixation of the ulna was performed if the surgeon determined reduction of the radius was adequate based on patient age [22]. Patients were followed with radiographs at regular intervals, initially at 2 weeks postoperatively, then approximately every 2–4 weeks. Primary implants were removed after radiographic evidence of healing at an average of 3–4 months after the index procedure. Technique for revision surgery was chosen by the treating surgeon and later implant removal was at their discretion.

## Statistical analysis

Descriptive statistics, including mean and proportion, were used to present the outcomes of our review.

## Results

Of the 485 patients treated with intramedullary implants for forearm fractures, six patients (1.2 %) with fractures with in situ IM implants were eligible for inclusion in this review (Table 1). There were 4 males and 2 females included, with a mean age of 10.6 years (Range 4–16 years) at the time of initial injury. All six patients had an injury to both forearm bones in the middle 1/3rd of the diaphysis. For patients 2, 3, and 6, surgery was performed within 48 h of injury. Patient 1 was taken to the OR 2 weeks after the injury, patient 5 1 month after injury, and patient 4, 9 days after injury, all after a loss of initial closed reduction was noted in clinic. Three patients (Patients 2, 3, and 6) had grade 1 open fractures, with patient 2 sustaining a segmental fracture of the radius and patients 2 and 6 developing ulnar nerve palsies at the time of injury that resolved over the course of follow-up. An open reduction was performed through the open wound in these three patients. Patient 4 required an open reduction at the initial surgery to successfully pass the IM implants. Another patient (Patient 5) required removal of his ulnar rod with retention of his radial implant 3 months after the initial surgery due to implant prominence and discomfort.

**Table 1:** Summary of patients with forearm fractures and in situ implants

Patient	Age (years)	Gender	Mechanism of injury	Other diagnosis	Open fracture	Bones fixed	Method of fixation	Open reduction	Nail:radial diameter
1	6	Female	Fall from height	None	No	Both	K-Wire	No	0.66
2	16	Male	Skateboarding	None	Yes	Both	TENS	Yes	0.48
3	4	Male	Playground	None	Yes	Ulna	K-wire	Yes	N/A
4	8	Female	Trip and fall	OI type III	No	Ulna	K-wire	Yes	N/A
	10	Female		OI type III	No	Ulna	K-wire	Yes	N/A
	14	Female		OI type III	No	Both	K-wire, TENS	No	0.59
5	13	Male	Soccer	None	No	Both	K-wire, TENS	No	0.40
6	14	Male	Skating	None	Yes	Both	TENS	Yes	0.58

Patient	Nail:ulnar diameter	Complications	Time to refracture (months)	Healed	Mechanism of refracture	Angulation	Treatment	Complications	Time to union (months)
1	0.66	None	5	Yes	Running, trip and fall	25	Rebending radial implant, replacement ulnar implant	None	1
2	0.52	Nerve injury	3	Yes	Skateboarding	34	Removal of nails, plate	None	2
3	0.57	None	4	Yes	Fall from bed	36	Replacement of nails	None	2
4	0.57	None	19	Yes	Fall from wheelchair	16	Casting	None	9
	0.57	None	7	Yes	Fall from wheelchair	49	Replacement of nails	Implant penetration	3
	0.57	None	45	Yes	Direct blow	12	Casting	None	7
5	0.33	Prominent implant	15	Yes	Snowboarding	4	Casting	None	3
6	0.62	Nerve injury	6	Yes	Skateboarding	51	Removal of nails, plate	None	2

All six patients suffered a fracture with in situ implants after radiographic confirmation of healing but before implant removal at a mean time of 13.0 months from index procedure (Range 3–45 months) and 11.1 months from healing (Range 0–43 months). Each sustained a second traumatic event that was similar in energy to the original mechanism leading to refracture. The mean angulation of the refracture was 28.4° (Range 4–51°). One patient (Patient 5) had a fracture distal to the radial implant that was adequately aligned. He was treated with closed casting without manipulation in the clinic and did not require a trip to the operating room for treatment.

**Fig. 1:**
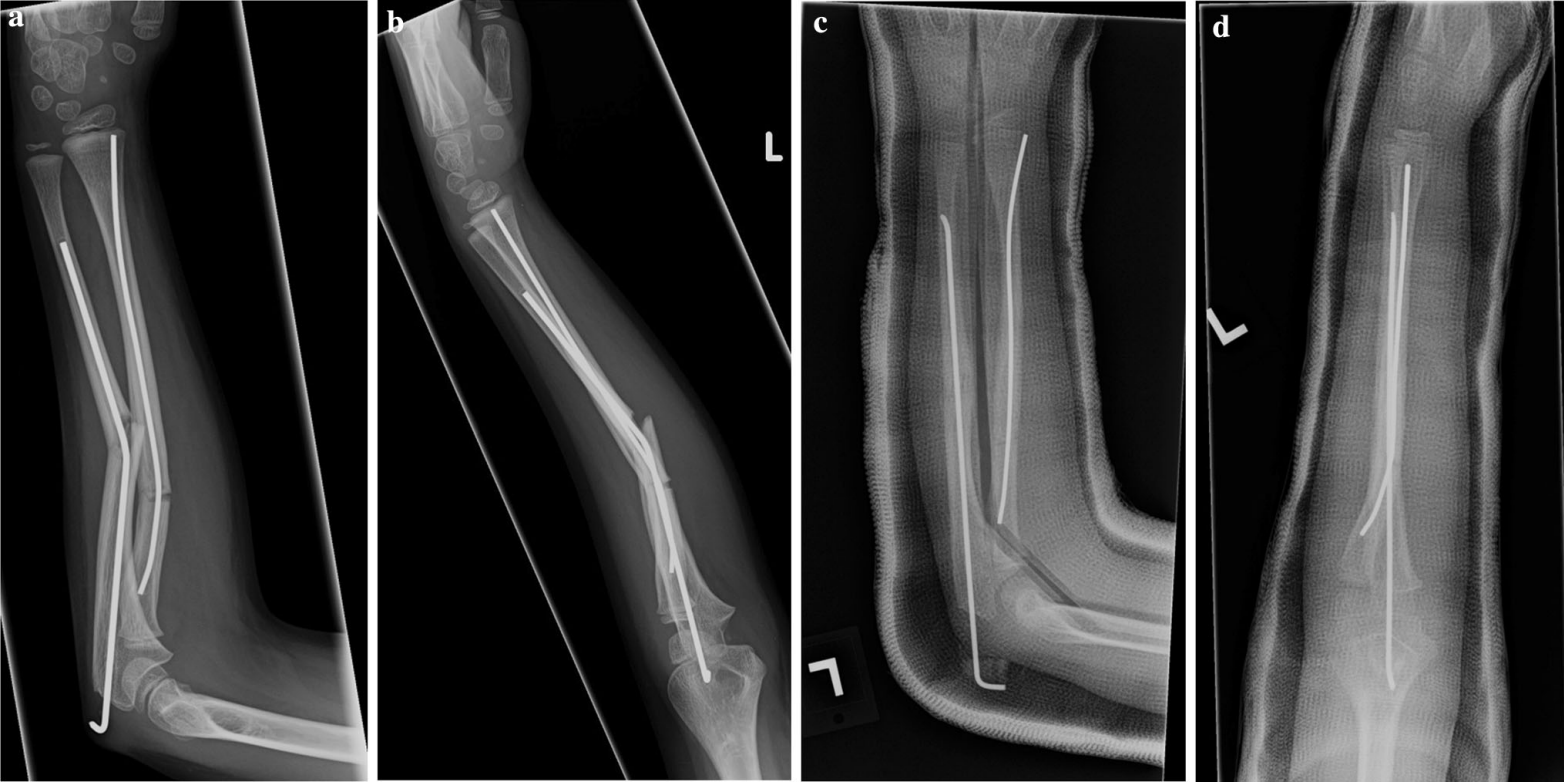
Anteroposterior (**a**) and lateral (**b**) radiographs of a 6 year old girl (Patient 1) who sustained a refracture after a trip and fall. She was treated with rebending of the radial implant and removal and replacement of the ulnar implant (**c**, **d**)

**Fig. 2:**
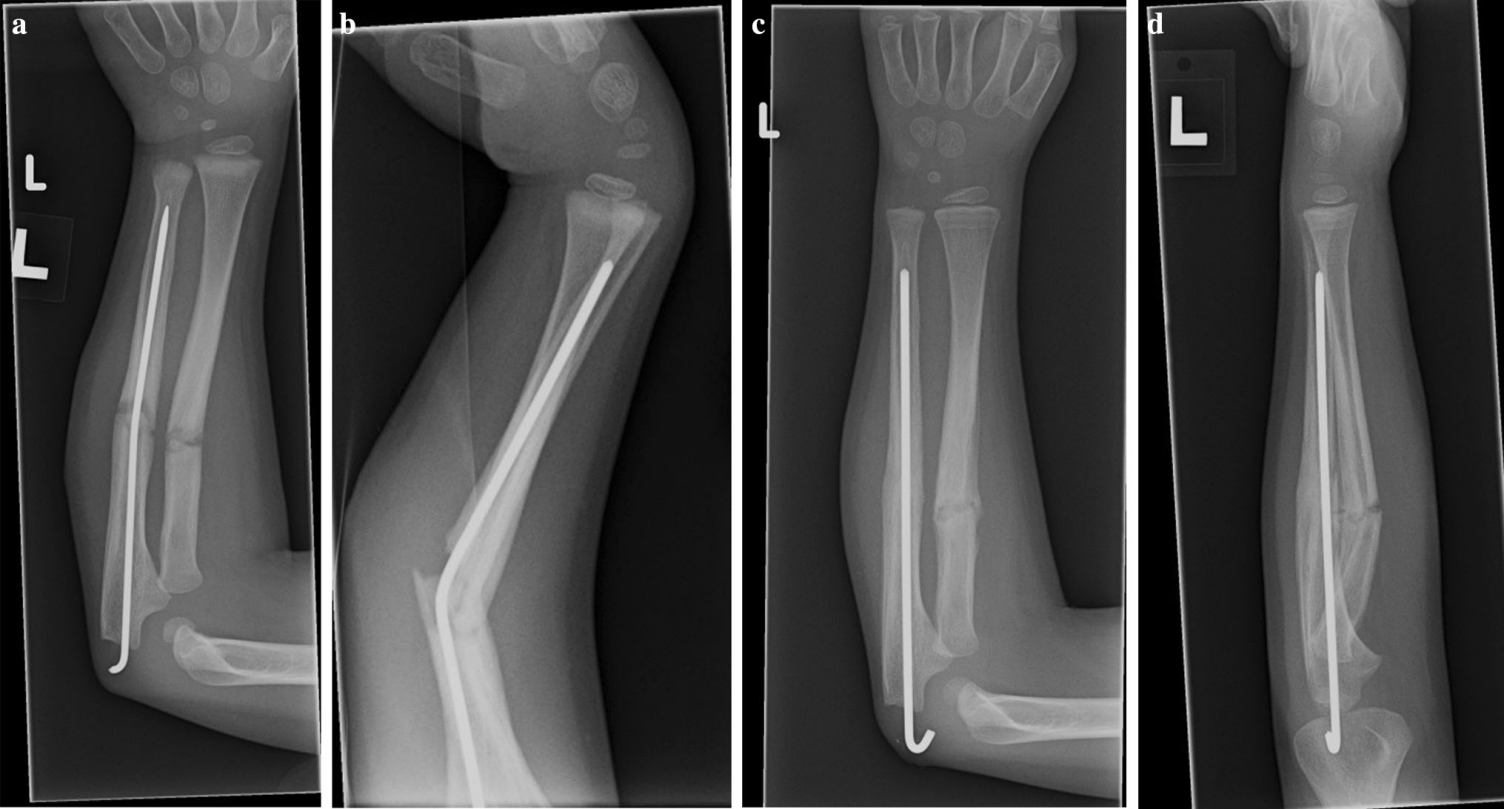
Anteroposterior (**a**) and lateral (**b**) radiographs of a 4 year old boy (Patient 3) who sustained a refracture from a fall from bed. He was treated with removal of his ulnar implant and introduction of a new nail (**c**, **d**)

**Fig. 3:**
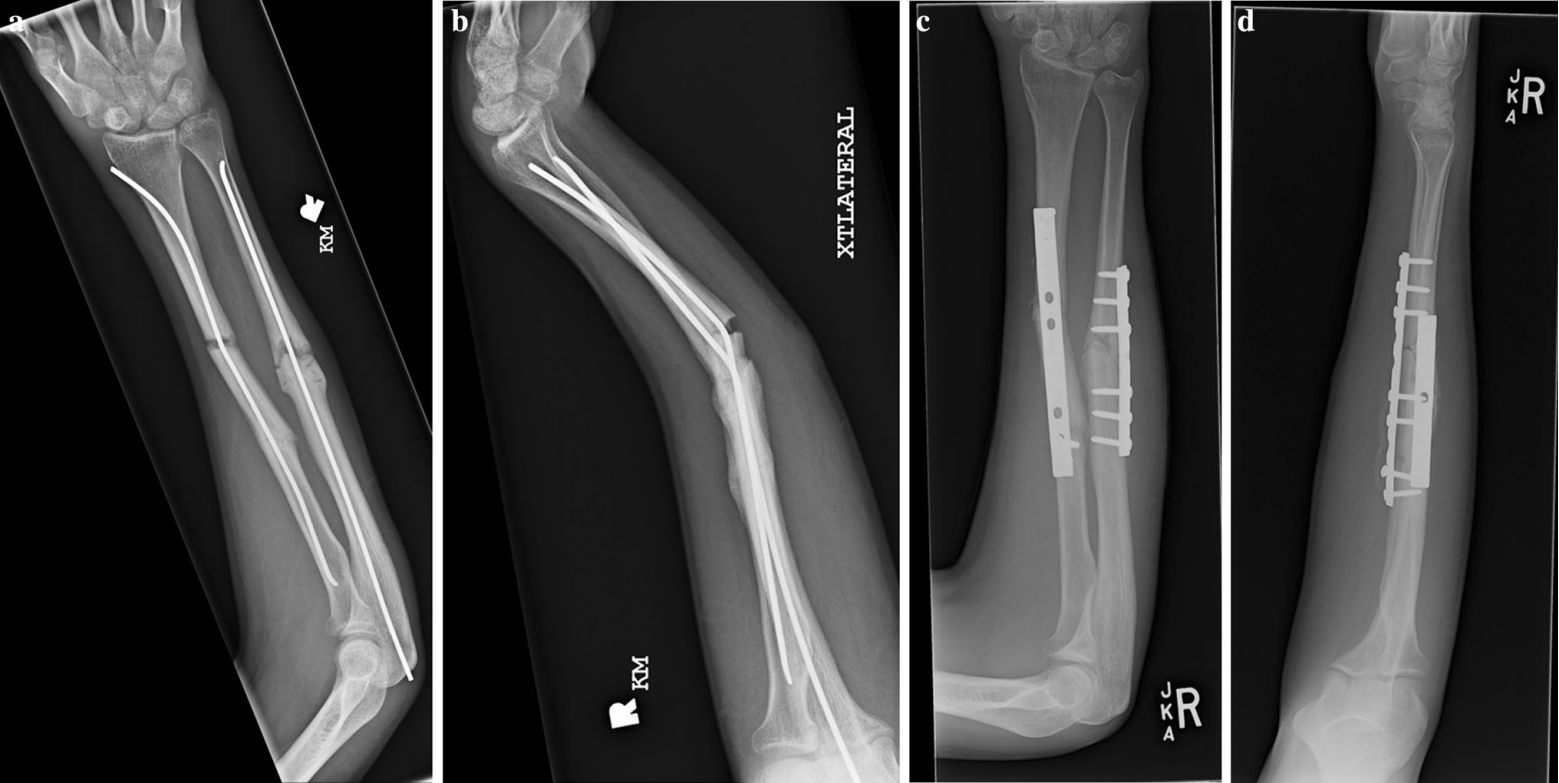
Anteroposterior (**a**) and lateral (**b**) radiographs of a 16 year old boy (Patient 2) who sustained a refracture while skateboarding. He was treated with removal of his implants and plating (**c**, **d**)

The remaining five patients (83 %) had unacceptable alignment of their fractures and returned to the operating room for a second surgical procedure. One patient (Patient 6) had an attempt at closed reduction under conscious sedation in the emergency department due to concern for skin tenting, but had continued unacceptable alignment of the fracture. The remaining four patients were taken to the OR without an attempt at closed reduction in the emergency room due to surgeon preference. One patient (Patient 1, Fig. 1) had successful closed bending of the radial implant under general anesthesia in the OR with residual deformity of the ulnar implant and subsequent replacement of the ulnar implant. Two patients (Patients 3, 4) had removal of their intramedullary implants with replacement of new nails (Patient 3, Fig. 2). Patient 3 had an attempt at closed reduction in the OR prior to implant exchange with continued unacceptable alignment and underwent single bone fixation of the ulna with acceptance of the residual radial deformity because of the patient’s age. Patient 4 underwent replacement without an attempt at closed reduction. One patient (Patient 2, Fig. 3) had removal of nails with plate osteosynthesis because of his age and skeletal maturity utilizing stacked 1/3rd tubular plates for both the radius and ulna and one patient (Patient 6) with stacked 1/3rd tubular plates for the ulna and 3.5 mm LC-DCP plate for the radius (Synthes, West Chester, PA ). Patients 2 and 6 did not undergo an attempt at closed reduction prior to plating.

Patient 4 was diagnosed with type III osteogenesis imperfecta (OI), and suffered three forearm refractures with in situ implants. Two of these refractures were minimally displaced and were treated with immobilization without reduction. The third fracture was treated with removal and replacement of nails in the OR because of unacceptable alignment without an attempt at reduction. This patient presented with penetration of the ulnar implant approximately 1.5 months after revision surgery, requiring a return to the OR for removal with retention of the radial implant.

All patients went on to uneventful healing of their injuries at a mean of 3.6 months from revision surgery. There were no other complications during the follow up period for any of the patients after their refracture or revision surgery.

## Discussion

In our series of patients with forearm fractures treated with IM implants, we identified six patients who suffered a fracture while implants were still in place. This represented approximately 1.2 % of the patients treated with IM implants during the study period. As we hypothesized, all patients went on to successful healing of their fractures, but only five of six patients required a second trip to the operating room for revision surgery.

The timing for removal of IM implants for pediatric forearm fractures has historically been based on the rate of refracture, which can be as high as 4–8 % in patients treated non-operatively [10, 11]. The initial investigations into the use of titanium elastic nails for pediatric forearm fractures highlighted the risk of refracture after implant removal [10]. In the series from Nancy, France, implants were buried and removed after an average of 4.25 months for their first 50 patients. After observing 3 refractures in this group, they altered their practice and began removing implants at 10 months to 1 year following the index procedure, after which no further refractures were seen. Due to this experience, intramedullary implants are typically left in place for at least 6–12 months prior to removal to provide mechanical protection against refracture [23]. However, refracture prior to explant of these IM devices is a rare complication that has been recognized and described.

There are case reports in the literature of refractures occurring before removal of the implants and their treatment. Mittal et al. reported on one case in a 14 year old boy with 2.0 mm titanium nails in place [12]. The refracture occurred after radiographic confirmation of healing and at 5 months after the index surgery. An attempt at closed reduction was made, but this resulted in breakage of the ulnar nail and ultimately the nails required replacement in the operating room for adequate realignment. Shahid et al. described another successful technique in a 10 year old girl who refractured 3 months after fixation [13]. They returned to the operating room and withdrew the nails a short distance without removing the nail completely so a straight portion of the nail crossed the fracture site, resulting in improved alignment. She went on to uncomplicated healing.

Muensterer and Regauer describe a 13 year old boy with 2.5 mm titanium nails who suffered a refracture with 21° of angulation 1 month after initial fixation [14]. This patient underwent successful closed reduction with rebending of the implants, and the nails were removed 5 months later after healing. They went on to test the mechanical properties of both titanium elastic nails and stainless steel nails bent to 21° in vitro. They determined that the force required for permanent deformation of previously bent nails decreased 37 %, and there was no evidence of metal fracture or fatigue after one cycle of reversed bending. None of the nails tested fractured after five cycles of bending and reversed bending. On the basis of their experience and results, they suggest that closed reduction and re-bending of in situ intramedullary implants is a mechanically viable option for forearm fractures with in situ implants.

The patients in our series were treated by several different methods, which include closed casting, removal and replacement of nails, in situ bending of nails, and removal of nails with plate osteosynthesis. There is currently no consensus as to the best method of treatment of forearm fractures with in situ IM implants. On the basis of our series, these differing methods are all viable options for treatment, and the surgeon should be able to expect that uncomplicated healing will occur with whatever method is chosen. This is in agreement with the previously published case reports. Based on this study and the current literature, it is our preference to treat these injuries with removal of bent implants and revision fixation in the operating room. Closed reduction and rebending of the initial implants maybe attempted initially as a temporizing measure in setting of skin tenting and soft tissue compromise; however residual deformity of retained implants may lead to suboptimal bony alignment. When performing revision fixation, a low threshold for open reduction is needed, as often percutaneous passage of new IM implants is difficult. In patients at or nearing skeletal maturity, plate fixation should be considered.

This study has several limitations. First, the retrospective design is subject to selection and treatment biases. Second, due to the very rare nature of this complication, a small number of patients were available for this review. As such, it is not possible to recommend a specific technique for the treatment of or to draw any conclusions about predictors of refractures with in situ implants. Generalizability of the results may be limited, as all patients were from a single, high volume pediatric tertiary care center. Finally, one patient in the series was diagnosed with OI, which could be considered separately. However, given the rare nature of fractures with in situ implants, this patient was included to provide further information about the complication. As most implants will be left in place long term for patients with OI, it is important to keep in mind that normal growth of the arm can make removal of implants difficult or impossible, and treatment of fractures with in situ implants may need to be adjusted accordingly.

In conclusion, refracture of the forearm in pediatric patients with IM implants in situ is a rare but recognized complication, occurring in approximately 1.2 % of patients treated with IM implants. Despite the numerous options for treatment of these injuries, all refractures in our series went on to uncomplicated healing following appropriate bony realignment and stabilization.
